# The Port Health Service

**Published:** 1957-04

**Authors:** D. T. Richards

**Affiliations:** Senior Port Medical Officer, Bristol


### THE PORT HEALTH SERVICE

BY

DR. D. T. RICHARDS

*Senior Port Medical Officer, Bristol*

"It is better to travel hopefully than to arrive . . ." R.L.S.

### I

### THE DEVELOPMENT OF PORT HEALTH LEGISLATION

The Port Health Service, serving both sea and air ports, is administered centra by a section of the Ministry of Health and locally by the Port Health Authority. is a service which receives little publicity. In fact, it is only when a case of smallpo for example, is discovered or overlooked at one of our sea or air ports that the pub hears of the existence of Port Health Departments.

The development of the Port Health Service is closely linked with the roma story of shipping and seamen. The Adriatic Republic of Ragusa first introduce system of maritime quarantine in 1377. Seafarers suspected to be suffering fr plague were isolated for 30 days at a place "distant from the harbour". This wa time when bubonic plague was devastating Europe. History associates Venice wi the 40-day period, or quarantenaria, which, partly for astrological reasons, beca accepted as the usual period of isolation for suspected seamen and seaborn merchandi

Since the introduction of quarantine in the fourteenth century each civilized mariti nation, in turn, introduced such a system. These national quarantine regulatio probably minimized, even if they could not entirely prevent, the entry of infectio diseases.

The first English quarantine regulations were drawn up in 1663 and provided f the isolation of suspected ships and their crews for 40 days in the Thames estua This did not, however, suffice to prevent the Great Plague of London which caus the loss of 70,000 lives from a population numerically smaller than that of pres day Bristol.

By the nineteenth century then was a large vested interest in quarantine measu and the associated lazar-houses. Quarantine procedures in each country, and so times from port to port in the same country, lacked uniformity. They were arbitrar often haphazard and extremely irksome and costly to trade and travel. The restrictio delays and hardships experienced by the eighteenth-century traveller are graphicall described in the memoirs of Rousseau and John Howard.

England, during this period was fast becoming the world's leading maritime natio The practice of preventive medicine made long sea voyages possible and, by controlli such diseases as scurvy, opened up the world to our ships. John Woodall (1569—164 an Elizabethan surgeon in the service of the East India Company, is credited with th discovery of lime or lemon juice as a cure for scurvy long before James Lind's treatis on the subject in 1753. Woodall recommended "the juice of vegetables and frui and when none can be had, oil of vitreol."

During Capt. Cook's expedition of 1772–75 a medical officer visited his shi entering Table Bay at Cape of Good Hope and inquired into the health of his crew being specially interested in smallpox. This must be one of the earliest port medica officer appointments. Cook's remarkable success in maintaining his seamen in goo health and free from scurvy was attributed by himself to an insistence on cleanlines ventilation, fresh water and fresh provisions, both animal and vegetable.

In the early nineteenth century new influences were at work. First, then was the rapid expansion of international trade coincidental with the Industrial Revolution. Our diminishing agriculture created a need for more imported food. Communications were speeded by the invention of steamships and railways (1810–1830). Thus, our maritime trading interests became much less tolerant of the losses and delays enforced in the name of quarantine in the world's ports. Increasing diplomatic pressure was exerted to abolish quarantine or render the measure more stable and uniform.

Secondly, the spread of Cholera from India to Russia, Europe and the United States of America increased the fear of imported epidemics but showed that the quarantine measures then in force were powerless to prevent the dissimination of cholera. It was realized that Cholera entered equally easily the closed ports of Greece and the open ports of Britain (Woolf, 1916).

Next, many authorities were proclaiming that disease was never transmitted by contagion but by cosmo-telluric influences coupled with accumulations of dirt and filth and that the proper defence against epidemics was sanitation, not quarantine. Koch did not discover the *vibrio Cholerae* until 1884 and there was no agreement before then within the medical profession as to how Cholera was contracted or could be prevented.

Finally, the harsh restrictions on ships coming from infected countries led to attempts at corrupt evasion such as the production of fraudulent Bills of Health. Epidemic outbreaks were concealed in order to avoid retaliatory measures.

Clearly the moment for international discussion and action had arrived. It was necessary that some degree of uniformity should be secured, particularly for such diseases as cholera, plague and yellow fever.

British opinion was anti-quarantine. The real argument against quarantine was put by Simon (1868) and has often been quoted: "A quarantine which is inaffective is a mere irrational derangement of Commerce, and a quarantine of the kind which ensures success is more easily imagined than realized. . . . The conditions which have to be fulfilled are conditions of national seclusion."

The first International Sanitary Congress was held in Paris in 1851. Twelve maritime nations were represented. The object of the negotiations was to reach agreement on preventive measures against cholera, plague and yellow fever, especially in the Mediterranean shipping zone. A measure of agreement was reached; the principle of quarantine was retained; international rules were drawn up providing uniformity in quarantine procedure; maximum and minimum quarantine periods were prescribed; lazarettes were henceforth to be hospitals rather than prisons; a restriction was to be enforced upon quarantine dues, which were in future to be uniform and not to be regarded as a source of revenue.

Thereafter a number of international sanitary conferences were held in order to bring into force effective preventive measures in the light of current advances in epidemiology. The last major conference took place in Paris in 1926 and on this occasion procedure was agreed which, very largely forms the basis of modern port health legislation. This 1926 Convention introduced a system for the control of typhus and smallpox, provisions were made for an improvement in the method of epidemiological reporting, and an international system for the control of rat infestation in ships was established.

These conventions were diplomatic instruments subject to formal ratification by signatory states. They were imperfect because even in questions concerning which international agreement was reached this agreement was sometimes withdrawn, and ratification was invariably delayed for years.

By the end of World War II it became apparent that the existing arrangements, together with the cumbersome diplomatic procedure involved, were unsatisfactory. Various countries had widely differing sanitary codes, and uniformity was still lacking.

The World Health Organization (1946), brought a new approach to the control of internationally transmissible diseases. W.H.O. is a specialized agency of the United

Nations Organisation, and in order to keep its work as free as possible from polit[illegible] influences, it is largely autonomous in matters of administration and finance.

In July, 1946, the Constitution of W.H.O. was drawn up and it was recogn[illegible] that one of the essential functions of the organization would be "to propose conv[illegible]tions, agreements and regulations and to make recommendations with respect [illegible] international health matters." Article 21 of the Constitution gives to the He[illegible] Assembly of W.H.O. "Authority to adopt regulations concerning sanitary [illegible] quarantine requirements and other procedures designed to prevent the internatio[illegible] spread of disease."

An important feature of the Constitution of this Organization is that Internatio[illegible] Sanitary Regulations *automatically* come into force for all members states after th[illegible] adoption by World Health Assembly. They no longer have to await ratification.

The idea of such a Health Agency is not a new one. It was originally advocated [illegible] Conference in 1874 and created at the Rome Convention in 1907 when the Of[illegible] Internationale d'Hygiene Publique was set up in Paris. But until the advent of Wo[illegible] Health Organization international health control was based mainly upon time-w[illegible] principles involving merely the erection of sanitary barriers. "Implicit in all th[illegible] strivings towards the foundation of an international health agency was not a wish [illegible] the general betterment of the health of the world, but the desire to protect cert[illegible] favoured (especially European) nations from contamination by their less favou[illegible] (especially Eastern) fellows. Half a century later the Constitution of the World He[illegible] Organization is a measure of the tremendous moral evolution which has made [illegible] impossible to accept as part of the national order the existence of preventable dise[illegible] and suffering over a large part of the habitable globe." (Howard-Jones, 1950.)

II

PORT HEALTH CONTROL—VESSELS ARRIVING

There are some 62 Port Health Authorities in England and Wales operating [illegible] International Sanitary Regulations. These Regulations, the outcome of three ye[illegible] study and discussion by an Expert Committee, were adopted by the World He[illegible] Assembly in October, 1952 and became binding on all member States of World He[illegible] Organization. Their object is ". . . to permit countries to take reasonable measures [illegible] protection against invasion by epidemic disease, while at the same time protecti[illegible] commerce and traffic between countries from excessive and often panic measur[illegible] tending to interfere seriously with international trade movement." (M. T. Morg[illegible] 1952.)

Under these Regulations the captain of a ship which arrives from a foreign port [illegible] required to ascertain the state of health of all on board and to sign a "Mariti[illegible] Declaration of Health". If the answers to the questions in the Declaration of Hea[illegible] are unsatisfactory, the ship is boarded by a Medical Officer before pratique is grante[illegible] It is still the duty of H.M. Customs to ascertain which ships require medical inspecti[illegible] and it is the officers of H.M. Customs and not the officers of the Port Health Author[illegible] who issue pratique, that is, permission for a ship to have free communication with t[illegible] shore.

The master of a ship which has had on board during its voyage a case, or suspect[illegible] case, of infectious disease or upon which there are other similar circumstances requir[illegible] the attention of the Medical Officer, must fly the flag signal "LIM" meaning [illegible] require the Port Medical Officer". This signal replaces the old signal "QQ" a[illegible] "QL" ("My ship is suspect" and "My ship is infected"), and suits the circumstan[illegible] for which "QQ". "QL" were intended, but has the additional advantage that it c[illegible] be used in other circumstances requiring the boarding of a ship by a Port Medi[illegible] Officer. Between sunset and sunrise the signal "LIM" must be flashed in the Mor[illegible] Code by lamp.

In other cases (i.e. when infectious disease is not suspected), if the ship arrives from a foreign port and is not engaged in regular packet-boat and excursion traffic with a port in France, Belgium or Holland, the flag signal "Q" is hoisted, or flashed in Morse Code. The "Q" signal, a single yellow flag, is a request for pratique.

These signals must continue to be shown until the ship is free from control, i.e. when a ship has been boarded, and cleared by the Port Medical Officer or the Customs Officer.

The flying of signals is not to be regarded as the discharge of the master's duties. Under the regulations he has still to prevent unauthorized persons from boarding his ship until it is freed from control. No person other than the officers of H.M. Customs and Immigration, the Pilot, or a member of the Port Health Staff may board a ship until it is freed from control, without the permission of the Port Medical Officer.

In consequence of an agreement reached between the Brussels Treaty Powers, certain continental ports are regarded as "Excepted Ports", and under normal circumstances ships arriving from these ports are exempt from certain of the requirements of these regulations. "Excepted Ports" are ports situated on the Atlantic coast of France, the coast of Belgium and of Holland.

A list of infected ports is kept up to date at the Port Health Office for the information and guidance of all concerned. For this purpose the port medical staff have available to them the excellent intelligence service of World Health Organization, The existence of the so-called quarantinable diseases at sea and air ports in every country is broadcast daily over a world-wide network from Geneva. This is digested and made available in a most accessible form in the Ministry of Health's Weekly Bulletin, which is forwarded to all Port Health Authorities.

In the regulations a distinction is drawn between "infectious diseases" and "quarantinable diseases". The former are defined as any infectious or contagious disease but do not include tuberculosis or the venereal diseases. The quarantinable diseases (sometimes referred to as the "Convention diseases") are cholera, typhus, plague, yellow fever, smallpox and relapsing fever.

Power is given to the Medical Officer to detain and examine any person suspected as suffering from an infectious disease or who has been exposed to infection. The patient may be removed to hospital and his clothing and effects disinfected. But in the event of the arrival of one of the "quarantinable diseases" certain additional measures are prescribed for the protection of the port. In all cases these are maximum measures and may not be exceeded; thus, in the case of smallpox the vaccination of all passengers and the entire ships' company may be carried out; surveillance, which does not unduly restrict the liberty of a contact, may be imposed for an appropriate period, or, when justified, the isolation of a suspect or a close contact may be insisted upon. The disinfection of water tanks and of dejecta before discharge from the ship must be carried out in the case of cholera; ship fumigation is essential when plague-infected rats are discovered; delousing must be undertaken when typhus occurs. Mails may in no circumstances be detained, disinfected or destroyed.

### III

### PORT HEALTH CONTROL—VESSELS IN DOCK

The routine inspection of all ships in port is an important phase of the work of the Port Health Inspectorate. The following matters receive special attention:

(*a*) Supervision of the living accommodation on ships; that is, the hygiene of crews' quarters. Immense improvements have taken place in the situation, type and condition of crews' quarters in recent years. "Slums at sea" are gradually disappearing and are being replaced by more commodious and comfortable cabins amidships. In this matter, Port Health Authorities and their Associations have played an important part, but there is still the need to educate some of the seamen to appreciate and ma[illegible] the best use of improved conditions.

Standards of accommodation which will make the seamen's life healthier and mo[illegible] comfortable have been laid down in the Merchant Shipping (Crew Accommodatio[illegible] Regulations, 1953. These Regulations give effect, in this country, to recommendatio[illegible] made at the Seattle International Maritime Conference (1946), and regulate the s[illegible] of berths, the amount of sleeping space per man, the maximum number of men p[illegible] cabin, the number of baths and washbasins, heating, lighting and ventilation, mess[illegible] and recreational facilities. But it must be pointed out that for many years, prior to t[illegible] issue of these Regulations, a great number of leading British shipowners, with t[illegible] health and well being of their crews in mind had already introduced considera[illegible] reforms. Often these were in advance of present statutory requirements, single-be[illegible] accommodation, for example, has featured for many years on the drawing boards [illegible] British marine architects.

(*b*) The inspection of ships for sanitary defects of original construction, for defe[illegible] due to wear and tear and for the existence of nuisances.

(*c*) The inspection of imported foodstuffs. All food cargoes are subject to inspecti[illegible] by officers of the Port Health Department. Meat or meat products when import[illegible] into this country must bear what is known as an "Official Certificate"; that is, a cer[illegible] ficate which testifies to the fact that the meat or meat product was, before importatio[illegible] examined by a competent authority in the country of origin, and that it has been pass[illegible] in accordance with criteria satisfactory to the Minister, and that all necessary precautio[illegible] for the prevention of danger to public health were taken in the preparation and pack[illegible] of the meat product. In the absence of this certificate, or should details on the certific[illegible] be absent or incorrectly filled in, the cargo is inadmissible and is liable to be re-export[illegible]

(*d*) The chemical and bacteriological examination of ships water supplies. Samp[illegible] of water from water boats and hydrants are regularly taken for analysis. Samples [illegible] water from ships in port are also submitted for examination, sometimes as the resu[illegible] of the occurrence of sickness on board, or following complaints from members of t[illegible] crew or Seamens' Union officials.

(*e*) In addition to the staff of Medical Officers, port health inspectors and cleric[illegible] staff, the Port Health Authority employs rodent operators whose duty it is to inspe[illegible] ships for rat infestation and to initiate measures for the control of the rat population [illegible] ships. In the past the discovery of swarms of rats when a ship's hatches were uncove[illegible] was not unusual. Today a thorough search in a large cargo ship seldom reveals mo[illegible] than a dozen rats and the majority of modern ships are so maintained that they a[illegible] almost rat-free. At one time the annual figure for rats recovered from vessels in t[illegible] Port of Bristol was numbered in thousands; nowadays this figure is usually well belo[illegible] 500. This important step forward is partly the result of rat-proofing in ships of mode[illegible] design, which has been said to create for the rat "a housing shortage, a water shorta[illegible] and a high rate of infantile mortality"; but mainly it is the result of internatio[illegible] legislation which, during the past 30 years, has prescribed that every foreign-goi[illegible] vessel must carry a valid "International Deratting Certificate" or "Deratting Exemp[illegible] tion Certificate", whichever is appropriate, both of these certificates being valid f[illegible] a period of six months, at the end of which time the vessel is subject to re-inspectio[illegible] and possibly fumigation with hydrogen cyanide or some other suitable rodenticide.

IV

### DISCUSSION

The United Kingdom is an extremely busy terminal for traffic arriving from coun[illegible] tries in which the quarantinable diseases are of major importance. The traffic enterin[illegible] Bristol, which is a leading port so far as this country's trade is concerned, is no ex[illegible] ception, goods from every country in the world being off-loaded at the quays [illegible] Avonmouth, Bristol City and Portishead Docks.

One of the primary duties of the Port Medical Officer is to assess the risk of importation of one of the quarantinable diseases into his area. In this he is guided by several factors. He is aware of the normal service to and from his seaport, connecting it with designated ports of the world. He is aware of the frequency of arrival of ships not on normal service. He is aware of the last day of report of a quarantinable disease at a foreign port—from which traffic is certain or likely to arrive at his own port. He has the further knowledge of the incubation periods of the "convention diseases" and of their most likely method of spread.

What is this risk?

Plague is an epizootic of certain rodent species, particularly of the black or ship rat. and in epizootic prevalences the disease tends to "spill over" into the human population. The vector is the rat flea. Sample rats are therefore regularly examined at the laboratories of the major ports. In the Port of Bristol fifty per cent. of the rats destroyed on ships in dock are submitted to the bacteriologist for an opinion. This precaution is worthwhile.

Neither rodent nor human plague has occurred in this country for many years, yet one must always keep in mind the experiences of Bristol during the First World War, when two cases of bubonic plague appeared as out-patients at the Bristol Royal Infirmary. Fortunately they were recognized, and plague-infected rats, which could only have entered the city via the port, were recovered from a rag and bone merchant's premises in the centre of the city, where the men worked. Such an incident leaves an indelible impression on the Port Health Services. The last occasion that an isolated case of clinical plague occurred on a vessel in Bristol was in 1932.

It is probably true to say that, provided our existing health services maintain their efficiency, the danger of the spread of human plague in Britain is not great; but the possibility of importation of this disease cannot be discounted.

Cholera is a disease of extreme rarity in this country. The classical method of spread is by the pollution of unpurified water supplies. The areas of endemic prevalence of this disease are geographically restricted. Is there a serious risk of the importation of cholera into this country through a sea port, and, if this should happen, what is the assessment of the danger of its spread in epidemic fashion? The clinical features of cholera are of such rapid development and dramatic intensity that, with our existing services, the risk of this disease being overlooked and allowed to enter the country at one of the ports is a small one; and the danger of its spread is, in fact, negligible.

Louse-born typhus has only occasionally been imported in recent years, and the human patient, once deloused is non-infectious. Now and again a person infested with lice is discovered at the port or in its vicinity, and the importation of typhus must always be regarded as a possibility. The high efficiency and ease of application of modern insecticides, however, provides the port health staff with an effective method of control over this disease, rendering the danger of its spread within our community very improbable.

There is no chance of spread of yellow fever, even in the unlikely event of the arrival of a fully established case at the port.

Smallpox, a disease feared and respected by every Port Medical Officer, is the outstanding danger in Port Health control. To some extent the Medical Officer is protected by the inevitable length of time which ships take to reach this country from endemic areas. The disease is widely distributed and highly infectious. It exists in countries with which the United Kingdom has a large volume of regular and occasional trade. It has been imported into the United Kingdom quite regularly over many years. An individual can obtain immunity to it through vaccination but the solidity of that immunity falls off with the passage of time, and a degree of immunity to smallpox in a person who has resisted several attempts at re-vaccination is a matter for conjecture.

To prevent the importation of smallpox and to help in limiting its spread, should importation be inevitable, makes the utmost demands on the skill and judgement [illegible] the port medical staff.

Having boarded a ship, the Medical Officer is guided by the Maritime Declarati[illegible] of Health; he is aware of epidemic conditions prevailing at the previous ports of ca[illegible] he has access to the medical log-book kept by the captain, and certain personal docu-ments of origin and health may be submitted to him. But not even the most caref[illegible] examination of documents, followed by medical examination, will identify the travell[illegible] who is incubating smallpox.

It is essential that the traveller should give every co-operation. This was made ve[illegible] clear on the occasion of the Brighton outbreak (1951). The primary case occurred in [illegible] patient who arrived in the United Kingdom from Pakistan and who felt ill within [illegible] hours of arrival, developing a rash not long afterwards. The patient did not se[illegible] medical advice. The consequences were serious.

But given the patients' co-operation smallpox is sometimes no easier to bring und[illegible] control. Two recent outbreaks in Scotland emphasize this point. The first was [illegible] Glasgow in 1941 where the primary case had sickened on shipboard with clinic[illegible] appearances leading to a provisional diagnosis of measles. The second instance, aga[illegible] at Glasgow, was in 1950, where the primary case sickened a few days after disembark[illegible]tion at London, was admitted to a Glasgow Hospital, and presented all the clinic[illegible] features of varicella. This is no reflection on the competence of those who made th[illegible] diagnosis on the clinical appearances of these two patients. The consequences of [illegible] mistaken diagnosis in the case of smallpox can be so disastrous that one should co[illegible]stantly keep in mind the immense help which can be given by the laboratory examina[illegible]tion of material derived from a patient, even in the earliest stages and even with th[illegible] most misleading clinical picture. The characteristic features of small pox can [illegible] greatly modified by successful vaccination, particularly during the period when sol[illegible] immunity is lost but partial immunity persists. In these circumstances the value [illegible] laboratory confirmation becomes relatively much greater.

Our dangers from the so-called quarantinable diseases are, to a large extent, th[illegible] danger of the importation and spread of smallpox, and to a lesser degree the danger [illegible] the importation of typhus.

### *BIBLIOGRAPHY*

*A History of Medicine:* Douglas Guthrie (T. Nelson & Sons, Ltd.).

"Contritutions to Medicine by Capt. James Cook, F.R.S., R.N.": W. R. Thrower, M.D[illegible] M.R.C.S. (*The Lancet*, 4th Aug., 1951).

*Chronicle of World Health Organization*, May 1951: Dr. P. Dorolle, Deputy Director-Gener[illegible]

*International Government:* L. S. Woolf (New York, 1916).

"*Origins of International Health Work*": N. Howard-Jones, O.B.E., M.R.C.S. (*B.M.J.*, 6t[illegible] May, 1950).

*Health at the Gateway:* E. W. Hope (University Press, Cambridge).

*The First International Sanitary Conference and Convention:* Neville M. Goodman (Asso[illegible] Sea and Airport Health Authorities, 1951).

"World Health Organization—International Sanitary Regulations No. 2": M. T. Morgan[illegible] C.M.G., M.C., M.D. (*Fluid Topics*, Vol. 3, No. 1, February, 1952).

